# Dielectric Barrier Discharge (DBD) Plasma Assisted Synthesis of Ag_2_O Nanomaterials and Ag_2_O/RuO_2_ Nanocomposites

**DOI:** 10.3390/nano6030042

**Published:** 2016-02-26

**Authors:** Antony Ananth, Young Sun Mok

**Affiliations:** Plasma Applications Laboratory, Department of Chemical and Biological Engineering, Jeju National University, Jeju 690-756, Korea; sebastianananth@gmail.com

**Keywords:** atmospheric pressure plasma, DBD, silver oxide, ruthenium oxide, nanomaterials, nanocomposite

## Abstract

Silver oxide, ruthenium oxide nanomaterials and its composites are widely used in a variety of applications. Plasma-mediated synthesis is one of the emerging technologies to prepare nanomaterials with desired physicochemical properties. In this study, dielectric barrier discharge (DBD) plasma was used to synthesize Ag_2_O and Ag_2_O/RuO_2_ nanocomposite materials. The prepared materials showed good crystallinity. The surface morphology of the Ag_2_O exhibited “garland-like” features, and it changed to “flower-like” and “leaf-like” at different NaOH concentrations. The Ag_2_O/RuO_2_ composite showed mixed structures of aggregated Ag_2_O and sheet-like RuO_2_. Mechanisms governing the material’s growth under atmospheric pressure plasma were proposed. Chemical analysis was performed using Fourier transform infrared spectroscopy (FTIR) and X-ray photoelectron spectroscopy (XPS). Thermogravimetric analysis (TGA) showed the thermal decomposition behavior and the oxygen release pattern.

## 1. Introduction

Silver oxide nanomaterials (Ag_2_O NMs) are widely used in catalysis [1,2], sensors [3], preparation of antimicrobial materials [4], drinking water-related applications [5], *etc.* It is an intrinsic p-type semiconductor with a band gap of around 1.5 eV. On account of its high catalytic activity under mild reaction condition, usage of Ag_2_O NMs is rapidly increasing in many chemical reactions. The efficiency of a material in an application can further be improved when it is mixed with other materials. Such carefully selected nanocomposite materials (two or more metallic or metal oxide structures having the dimension of nanoscale) having excellent individual properties exhibit new and unique characteristics without affecting each other. For example, Lee *et al.* [6] have documented that Ag_2_O/RuO_2_ composite exhibits higher capacitance as compared to its individual performances. Ruthenium (IV) oxide (RuO_2_) itself is an excellent candidate material for catalysis, field emission displays, fuel cells and supercapacitor applications [6,7,8]. There are varieties of methods such as wet chemical [9], biological [10], thermal deposition [11], thin film-based and recently plasma-mediated routes [12,13,14,15,16,17] to prepare nanomaterials are currently used. The main advantage of applying plasma-based techniques to synthesize nanomaterials is the possibility of controlling the growth, surface chemistry and surface morphology. For instance, plasma-mediated synthesis does not require stabilizer molecules to prevent the aggregation (steric stabilization) which is commonly encountered in wet chemical synthesis. This is because the growth of material and the control of aggregation are entirely driven by the physical properties such as electrostatic repulsion, electric potential and conductivity (both electrical and thermal) [18]. The surface chemistry (metallic and oxide formations) is mostly decided by the composition of the feed gas used for the generation of plasma. Depending on the gas composition, the density of the active species such as electrons, ions and radicals vary and its interaction with the nucleating particles decides the chemical nature of the surface. The drawbacks of plasma techniques may be electrical safety of high voltage systems and difficulty in mass production.

Unlike other vacuum-based (for example, chemical vapor deposition (CVD), plasma enhanced CVD, *etc.*) or high-temperature plasma techniques, the dielectric barrier discharge (DBD) plasma-based method is non-thermal in nature, works at atmospheric pressure, and can operate at relatively low energy input, which facilitates the production of nanomaterials with unique structural features [8]. In addition, the construction of DBD plasma reactor is relatively easier than any other plasma techniques and the DBD plasma gives reproducible results. In our previous reports [8,14], RuO_2_ NMs were synthesized in a DBD plasma reactor, and it was found that the gas composition and the substrate materials used for the growth of NMs played main roles in determining the morphology of the NMs. To our knowledge, the investigation on the growth of composite materials having two different physicochemical properties in the presence of atmospheric pressure plasma is scarce in the literature [18,19]. With this background, this work deals with the syntheses of Ag_2_O NMs and Ag_2_O/RuO_2_ nanocomposite materials in the presence of DBD plasma. Investigation on the surface morphological control, growth mechanism and surface chemical analyses of the plasma-assisted materials would help to understand the importance of this method. The Ag_2_O/RuO_2_ composite material was chosen based on its potential environmental applications.

## 2. Results and Discussion

### 2.1. X-Ray Diffraction Study for Structural Analysis

**Figure 1:**
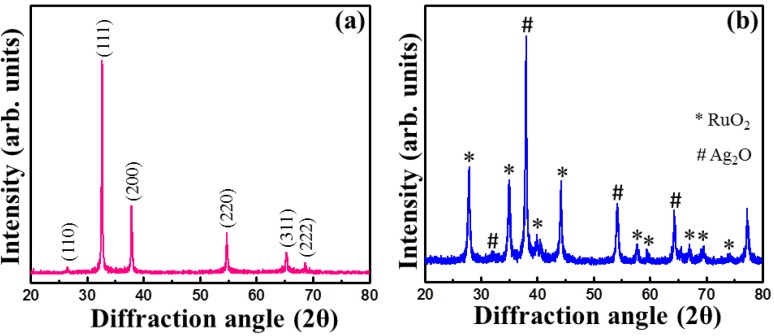
X-ray diffractograms of (**a**) Ag_2_O nanomaterials (NMs); and (**b**) Ag_2_O/RuO_2_ nanocomposite. Arb. units stand for arbitrary units.

The X-ray diffraction (XRD) spectrum of Ag_2_O NMs is given in Figure 1a. The peaks observed at the diffraction angle (2θ) 26.5°, 32.75°, 37.9°, 54.8°, 65.3° and 68.7° correspond to (110), (111), (200), (220), (311) and (222) set of lattice planes (cubic structure), respectively [JCPDS card No. 76-1393]. The spectrum did not contain any peaks corresponding to impurities or metallic forms, thus showing the Ag_2_O NMs of high purity [20]. The high intense peak (111) may refer to the arrangement of lattice atoms in an ordered structural fashion. The XRD spectra in Figure 1b shows the pattern corresponding to Ag_2_O/RuO_2_ nanocomposite, in which the Miller indices representing Ag_2_O and RuO_2_ are marked with (**#**) and (*). Slight shift in the diffraction peak (200), (220) and (311) of Ag_2_O was noted at the 2θ angle 38.21°, 54.26° and 64.5°, respectively. This kind of shift (decrease or increase) in the diffraction angle results from the difference between Ag_2_O and RuO_2_ lattice constants and lattice strain [21]. Moreover, the intensity of (111) peak decreased to a great extent and (200) increased, which may indicate a structural change (especially it may be due to the morphology, since the XRD peak intensity is closely connected with crystal morphology [22]). In comparison, the peaks corresponding to rutile type RuO_2_ were clearly observed without any shift [9].

### 2.2. Surface Morphology and Elemental Analysis

**Figure 2:**
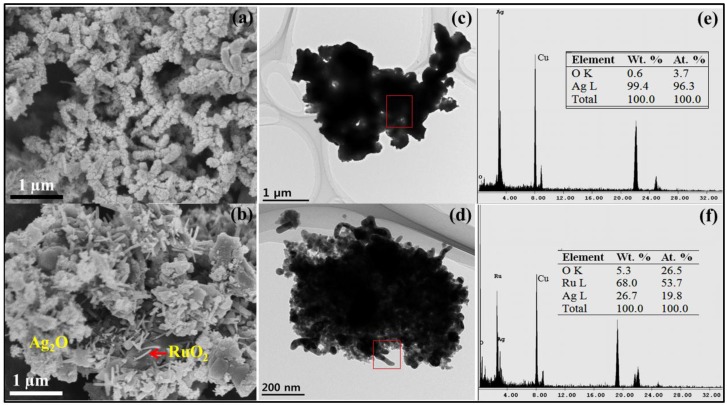
Field emission scanning electron microscope (FESEM) surface morphological images of (**a**) Ag_2_O nanomaterials (NMs); and (**b**) Ag_2_O/RuO_2_; high resolution transmission electron microscope (TEM) images of (**c**) Ag_2_O NMs; and (**d**) Ag_2_O/RuO_2_; and energy dispersive X-ray spectroscopy (EDX) spectra with the elemental composition for (**e**) Ag_2_O NMs; and (**f**) Ag_2_O/RuO_2_.

Figure 2a,b show the field emission scanning electron microscope (FESEM) surface morphological images of the plasma-synthesized Ag_2_O and Ag_2_O/RuO_2_ nanocomposite powders, respectively. The Ag_2_O NMs exhibited bundles of spherical nanoparticles (with particle diameter < 50 nm) whereas the Ag_2_O/RuO_2_ composite showed the mixed structures of RuO_2_ nanosheet and aggregated Ag_2_O. The high resolution transmission electron microscope (TEM) images also confirmed the above observation (Figure 2c,d). The elemental composition (atomic percentage) measured by energy dispersive X-ray spectroscopy (EDX) (Figure 2e) for Ag_2_O consisted mainly of Ag (96.3%) and O (3.7%). The peaks corresponding to Ag_2_O/RuO_2_ nanocomposite consisted of Ag (19.8%), Ru (53.7%) and O (26.5%) (Figure 2f). The other peaks found in the spectrum refer to copper and carbon of the TEM grid. For information, the EDX analysis gives the information on the elements present in the given sample, but the exact content of each element in the sample cannot be determined by this technique because the elemental composition depends largely on the scan area.

### 2.3. Particle Size Analysis

**Figure 3:**
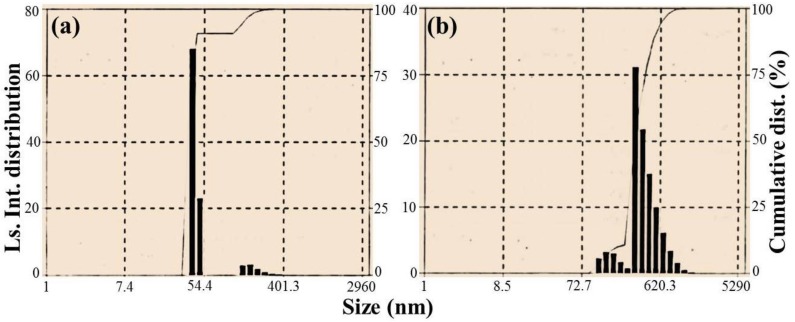
Particle size distributions of (**a**) Ag_2_O NMs (b); and (**b**) Ag_2_O/RuO_2_ nanocomposite. Ls. Int. distribution stands for light scattering intensity distribution.

From particle size analysis, size distribution of nanomaterials in an aqueous media can be obtained. This property is of particular importance, considering the applications such as photocatalysis, anti-microbial studies, *etc.* Particles of different size exert different Brownian motion in solution, and thus, when the particles are illuminated by a beam of light, the scattered light fluctuates in response to the individual particles. This fluctuation is monitored and using photon detection method, the particle size distribution is obtained. Particle size analyses were performed for Ag_2_O and Ag_2_O/RuO_2_ nanocomposite, and the results are shown in Figure 3 where the size (nm) of the nanomaterials *versus* light scattering distribution (probability) along with the cumulative distribution is represented. The cumulative distribution refers to the probability that the particle size is less than or equal to a particular size, *i.e.*, accumulated probability up to a particular size. The average hydrodynamic diameters (Stokes diameter) of Ag_2_O and Ag_2_O/RuO_2_ were found to be 44.2 and 485 nm, respectively. Since the Ag_2_O NMs showed uniform growth and morphology, more number of particles was observed within 100 nm range. The particles with larger size, especially in Ag_2_O/RuO_2_ composite may reflect the RuO_2_ length. Besides, the Ag_2_O NMs did not grow uniformly and exhibited more aggregation in the composite, resulting in bigger size distribution. Average size of the Ag_2_O and Ag_2_O/RuO_2_ nanocomposite obtained from the particle size analysis agreed well with the morphological features obtained by FESEM.

### 2.4. Effect of NaOH Concentration on the Morphology and Growth Mechanism

**Figure 4:**
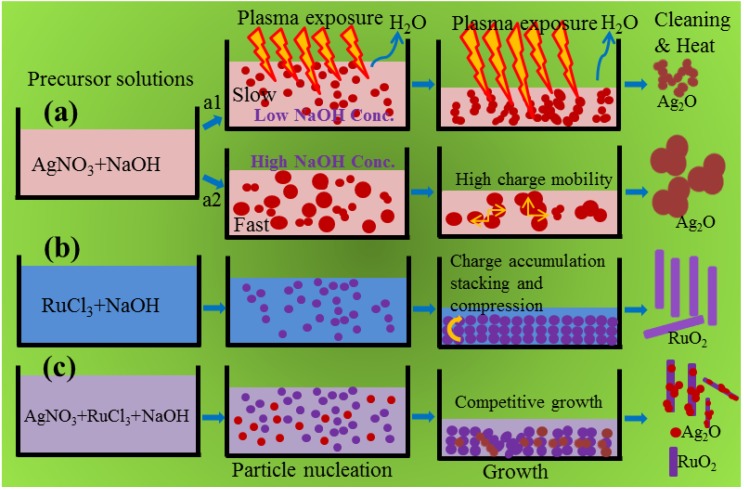
The proposed growth mechanisms for (**a**) Ag_2_O and (**b**) RuO_2_ nanomaterials and (**c**) Ag_2_O/RuO_2_ nanocomposite in the presence of plasma. The **a1** and **a2** represent the Ag_2_O growth at 0.5 and 1.0 M NaOH concentration.

**Figure 5:**
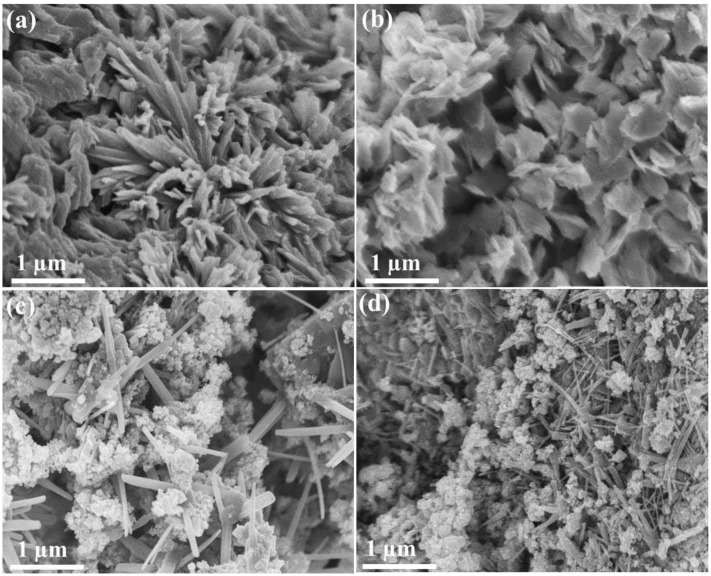
Morphology of Ag_2_O NMs obtained with an NaOH concentration of (**a**) 0.75 M; and (**b**) 1.0 M; and morphology of Ag_2_O/RuO_2_ nanocomposite obtained with an NaOH concentration of (**c**) 0.75 M; and (**d**) 1.0 M.

In the previous study [18], it has been reported that materials having higher electrical and thermal conductivities (for example, CuO) usually produce spherical particles in the presence of DBD plasma. Initially, supersaturation of the solution is attained as a result of the evaporation of water due to the heat generated by plasma. Then the accumulation of charged species on the surface of the growing particles favors the growth towards the oppositely charged electrode. Since the incoming electrons are highly mobile on the entire surface of the nucleated Ag_2_O species (Ag_2_O and CuO band gap values are almost similar, and thus the Ag_2_O follows similar growth pattern to CuO NMs), the alternating voltage does not produce any structural growth specifically. This is schematically proposed in Figure 4a (a1). In such cases, changing the NaOH concentration (pH increase) would be an alternative rather than altering the plasma parameters. Once the supersaturation is attained, the growth speed (slow or fast) of the nucleated particles may depend on the NaOH concentration. If the NaOH concentration is increased, attainment of zero charge molecules and formation of hydrated hydroxides starts earlier due to more OH^−^ ions from NaOH, which may result in bigger particles of different morphologies (Figure 4a (a2)). The mechanism was found suitable by observing the FESEM surface morphology of the Ag_2_O NMs obtained with 0.75 and 1.0 M NaOH concentrations, respectively (Figure 5a,b). When the concentration of NaOH was increased from 0.75 to 1.0 M, the length and thickness of the particles also increased, resulting in a flower-like and leaf-like morphology, respectively. On the other hand, the synthesis of RuO_2_ NMs (band gap of about 2.5 eV) by DBD plasma resulted in one dimensional structure such as nanosheets (see Figure S1b in Supplementary Materials) due to the potential gradient inside the reactor [15]. Accumulation of static electric charges results in the stacking and compression of the nucleated particles in response to the created electric potential, leading to sheet-like structures (Figure 4b). On the other hand, the length of the RuO_2_ nanosheet and the aggregation behavior of Ag_2_O were influenced to a great extent in the Ag_2_O/RuO_2_ nanocomposite when increasing the NaOH concentration (Figure 4c and Figure 5c,d). The nanocomposite exhibits mixed structures due to the competition between the nucleation and growth of the starting materials (one particle growth may suppress or hinder the other). It is learnt that the uniform growth with specific morphological features could be obtained if the NMs are prepared separately.

### 2.5. Bulk and Surface Chemical Analysis

**Figure 6:**
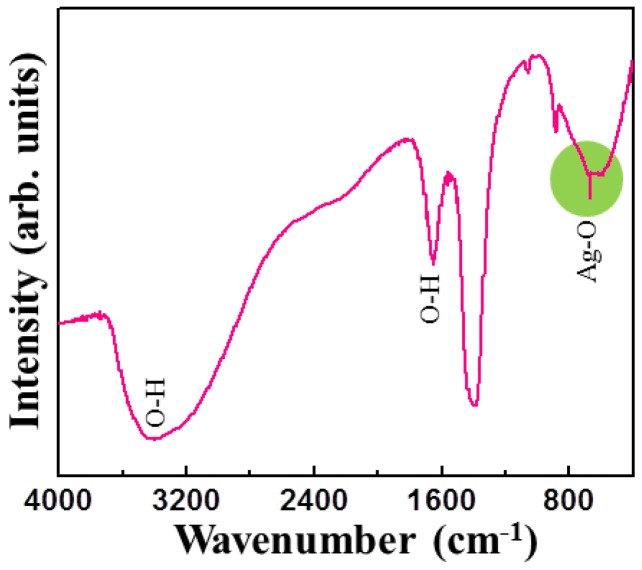
Fourier transform infrared spectroscopy (FTIR) spectrum of Ag_2_O NMs.

Figure 6 shows the Fourier transform infrared (FTIR) spectra of the Ag_2_O NMs. The Ag_2_O/RuO_2_ nanocomposite exhibited almost similar spectrum and thus not shown here. The functional groups corresponding to Ag_2_O nanoparticles (Ag–O bonds) were observed around 550 and 720 cm^−1^ (highlighted) referring to its transverse and longitudinal optical phonon vibrational frequencies, respectively [11,23,24]. The signatures at 1638 and 3400 cm^−1^ correspond to the vibration of OH. The metal-oxygen vibrations for Ag–O and Ru–O in Ag_2_O/RuO_2_ nanocomposite were observed between 650 and 800 cm^−1^. In this case, the identification of the peaks corresponding to Ag–O and Ru–O seems difficult since the composite exhibited identical FTIR spectra as observed for Ag_2_O.

**Figure 7:**
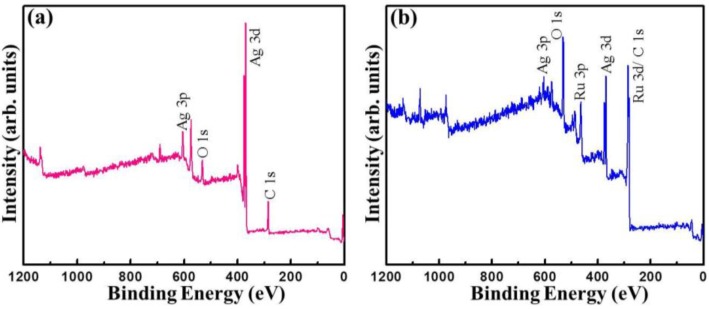
X-ray photoelectron spectroscopy (XPS) survey spectra of (**a**) Ag_2_O NMs (b); and (**b**) Ag_2_O/RuO_2_ nanocomposite.

**Figure 8:**
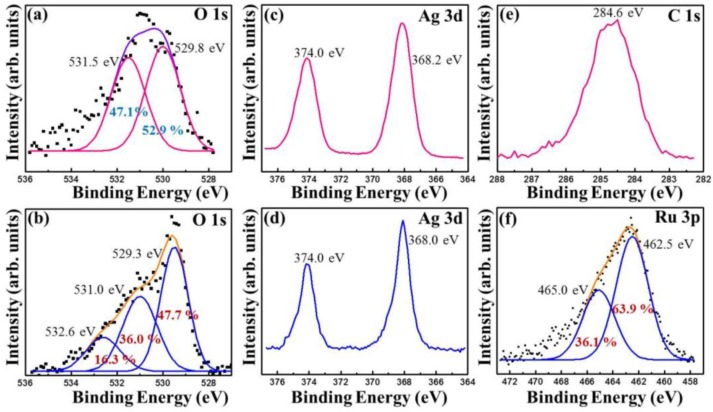
High-resolution core level XPS spectra of (**a**) O 1s in Ag_2_O; (**b**) O 1s in Ag_2_O/RuO_2_ nanocomposite; (**c**) Ag 3d in Ag_2_O; (**d**) Ag 3d in Ag_2_O/RuO_2_ nanocomposite; (**e**) C 1s in Ag_2_O; and (**f**) Ru 3p in Ag_2_O/RuO_2_ nanocomposite.

In order to confirm the presence of chemical functional groups in a detailed manner, the X-ray photoelectron spectra (XPS) of Ag_2_O NMs and Ag_2_O/RuO_2_ nanocomposite were taken and given in Figure 7a,b. The Ag_2_O spectra showed the presence of Ag (as 3d and 3p orbital splitting), oxygen and adventitious carbon signals [25,26]. The Ag_2_O/RuO_2_ nanocomposite exhibited the presence of ruthenium, silver and oxygen. The high resolution core level XPS spectra of O 1s, Ag 3d, C 1s and Ru 3p spectra corresponding to the samples Ag_2_O and Ag_2_O/RuO_2_ are given in Figure 8. The O 1s spectrum of Ag_2_O contains two species at the binding energy 531.5 and 529.8 eV referring to oxygen from adsorbed water and Ag_2_O [27,28,29,30,31], which are noted with its composition (atomic %). The O 1s spectrum of Ag_2_O/RuO_2_ contains one more peak corresponding to RuO*_x_* in addition to the above (Figure 8b) [32]. The presence of other elements were found out by Gauss-Laurentian peak fitting program and listed in Table 1. The Ag spectra of the Ag_2_O and Ag_2_O/RuO_2_ nanocomposite consisted of a doublet peak at the binding energy 368.2 eV (3d_5/2_) and 374 eV (3d_3/2_) with the doublet separation of 5.8 eV (Figure 7c,d). The Ru 3p spectrum of Ag_2_O/RuO_2_ contains two regions corresponding to RuO_2_ and higher oxides of ruthenium (RuO*_x_*_,_ where *x* = 3, probably) [33].

### 2.6. Thermal Study

The thermo-gravimetric analysis (TGA) technique is fundamentally used to track the changes in physical (phase transformation, crystallization) or chemical (redox reactions) process that occurs when heating a material. The decomposition reaction of Ag_2_O and Ag_2_O/RuO_2_ in the presence of N_2_ atmosphere can be expressed by the following equations. It is assumed that the sample contains stoichiometric oxygen and the weight loss is proportional to the amount of oxygen present originally.

Ag_2_O → 2Ag + 0.5 O_2_ (1)

Ag_2_O/RuO_2_ → 2(Ag/Ru) + 1.5 O_2_ (2)

The oxygen loss associated with the decomposition is as follows:

(3) $$\Delta W=\frac{\text{Atomic or molecular weight of oxygen}}{\text{Molecular weight of metal oxide}\left(s\right)}\text{Actual weight of metal oxide}\left(s\right)$$

**Figure 9:**
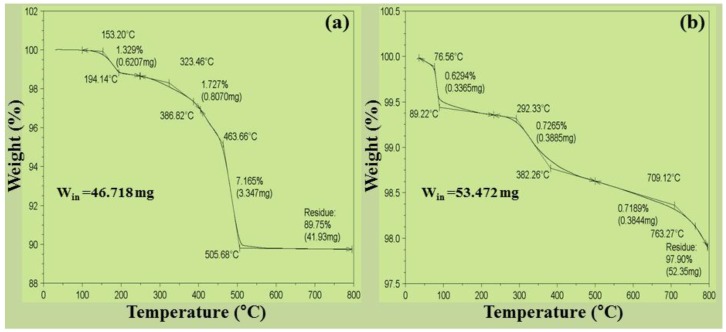
Thermogravimetric analysis (TGA) thermogram of (**a**) Ag_2_O NMs and (**b**) Ag_2_O/RuO_2_ nanocomposite (b). W_in_ : initial weight of the sample.

Figure 9a shows TGA thermogram of Ag_2_O NMs. It showed two stage decomposition or transformation starting at 194.14 °C and 386.82 °C, which could account for the decomposition of water (and also AgO) and the conversion from Ag_2_O to metallic Ag [34] with the activation energy of 283.4 kJ·mol^−1^ at the standard atmosphere [35]. Since the decomposition was rapid, about 10.25 wt % of the sample was lost at the end of the reaction. According to the above equations, the sample contains initially 6.89 wt % oxygen and final loss amounts to 6.19 wt %. This loss was observed up to 505.68 °C and beyond it the sample exhibited molten salt or ionic liquid [36] behavior with small percentage of oxygen (0.7%, see Table 2). But the Ag_2_O/RuO_2_ nanocomposite (Figure 9b) showed multi-stage decomposition phenomena. Initial water removal was observed until the temperature reached at 150 °C, followed by maximum Ag_2_O decomposition at around 382.26 °C and eventually conversion into metallic Ru formation above 700 °C with mass loss of 2.1 wt %. On considering the oxygen loss, it exhibited a gradual decomposition with its initial concentration of 13.149 wt % to the final loss 12.873 wt %. The Ag_2_O/RuO_2_ nanocomposite showed a steady oxygen release and maximum decomposition to metallic nature.

## 3. Experimental Section

### 3.1. DBD Plasma Reactor Setup

The NMs were prepared in a self-designed, rectangular box type DBD plasma reactor [8,15], the photographic image of which is given in Figure S2. In brief, two electrodes (4 cm wide and 15 cm long) acted as high voltage and ground electrodes which were covered with glass dielectric of 1.5 mm thickness. The DBD plasma reactor prepared as above was fixed in an acrylic chamber with gas inlet and outlet. The discharge gap (distance between high voltage and ground electrodes) was fixed at 1.5 cm and the nanomaterial precursor solution was kept inside. Alternating current (AC) high voltage (operating frequency: 400 Hz) was applied across the electrodes to generate the plasma. Since argon was used, plasma consisting of numerous filamentary microdischarges was readily created despite the large discharge gap.

### 3.2. Material Synthesis

Precursor chemicals such as AgNO_3_ (MW 169.87 g mol^−1^, Sigma-Aldrich, St. Louis, MO, USA), RuCl_3_·*x*H_2_O (Sigma-Aldrich, St. Louis, MO, USA) and NaOH (Shinyo Pure Chemical Co., Osaka, Japan) were used as received. Exactly 0.5 M AgNO_3_, RuCl_3_·*x*H_2_O and NaOH solutions were prepared separately. For the preparation of Ag_2_O NMs, 500 µL of the precursor solution was mixed with 500 µL NaOH, agitated for 2 min, spread on the glass substrate and kept inside the DBD plasma reactor [14]. For Ag_2_O/RuO_2_ nanocomposite, 500 µL respective precursor solutions and 1 mL NaOH were used. Gas purging with Ar gas was done for 15 min and the solution was exposed to plasma. The input power was maintained at 38.20 W for all the samples. The input power was measured by a digital power meter (Model WT200, Yokogawa, Tokyo, Japan). Depending on the sample dryness inside the plasma region, the discharge voltage fluctuated in the range of 16–20 kV, despite the same input power. The temperature inside the reactor was found to be varying from room temperature at starting to 70–76 °C at the end of the reaction. According to our previous study, conventional wet chemical synthesis produces aggregated spherical type of NMs [9]. At the end of 3-h plasma reaction, the solid powder was collected and washed repeatedly with deionized water. Heat treatment was performed in a furnace at 200 °C for 24 h.

### 3.3. Material Characterization

Crystalline nature of the materials was studied using an X-ray diffractometer (D/Max Ultima III, Rigaku Corp., Tokyo, Japan) fitted with a monochromatic Cu K_α_ radiation (wavelength, λ = 0.154 nm) operated at 40 mA and 40 kV. The surface morphology was observed by an FESEM (JEM 1200 EX II, JEOL, Tokyo, Japan) and TEM. The elemental composition and the nanomaterial’s size were analyzed using an EDX (Model: R-TEM, CM200-UT, Philips, Ventura, CA, USA) and a particle size analyzer (ELS8000, Otsuka Electronics, Osaka, Japan), respectively. FTIR spectroscopy (IFS 66/S, Bruker, Bremen, Germany) was used to study the chemical functional groups. Chemical nature of the surface was also characterized by using an XPS (ESCA 2000, VG Microtech, East Grinstead, UK) with monochromatic Mg K_α_ X-ray radiation (1253.6 eV) operated with a 13 kV and 15 mA excitation source. TGA was carried out to study the thermal decomposition behavior of the prepared NMs. The samples were heated at a ramping rate of 10 °C·min^−1^ and simultaneous weight loss was recorded precisely (Q50 Analyzer, version 20.10, TA instruments, New Castle, DE, USA).

## 4. Conclusions

The DBD plasma-mediated syntheses of Ag_2_O NMs and Ag_2_O/RuO_2_ nanocomposite were investigated. Both materials exhibited crystalline nature with a slight shift in the diffraction angle for Ag_2_O in the Ag_2_O/RuO_2_ nanocomposite. The Ag_2_O exhibited unique morphology of spherical bundles whereas the composite showed the mixture of RuO_2_ nanorod and Ag_2_O spherical aggregates. The Ag_2_O morphology was significantly altered by changing the concentration of NaOH. The surface chemical analyses by XPS revealed that the sample consisted mainly of Ag_2_O. The ruthenium in the Ag_2_O/RuO_2_ nanocomposite was in two oxide forms such as RuO_2_ and RuO*_x_*. The thermal study showed a fast transformation of Ag_2_O into metallic Ag in the temperature range of 200–500 °C. But the Ag_2_O/RuO_2_ nanocomposite exhibited gradual decomposition without major change in the initial weight until 700 °C. This nanocomposite may exhibit superior redox properties even at high temperature and thus it would serve as a potential candidate in many applications.

## Figures and Tables

**Table 1:** Results of the X-ray photoelectron spectroscopy (XPS) analysis.

Binding Energy (eV)	Species Assignment	Reference
284.6	Carbon	[25]
368.2, 374.0, 529.3, 529.8	Ag_2_O	[27,28,29,30,31]
462.5	RuO_2_	[32]
531.5	Oxygen from adsorbed water	[31]
531.0, 465.0	RuO*_x_*/Ru	[33]
532.6	Diffused oxygen atoms	[26]

**Table 2:** Amount of oxygen before and after Thermogravimetric analysis (TGA) analysis.

Sample	Initial Sample Mass (mg)	Initial O_2_ Mass (mg)	Final Sample Mass (mg)	Lost O_2_ Mass (mg)	Remaining O_2_ Mass (mg)	Remaining O_2_ Mass (%)
Ag_2_O	46.718	3.22	41.93	2.89	0.330	0.700
Ag_2_O/RuO_2_	53.472	7.031	52.35	6.883	0.148	0.276
^*^ RuO_2_	42.988	10.388	40.84	9.822	0.566	1.192

* Supplementary Materials.

## Supplementary Materials

The following are available online at http://www.mdpi.com/2079-4991/6/3/42/s1.
