# Supplementary material for: Dielectric Barrier Discharge (DBD) Plasma Assisted Synthesis of Ag2O Nanomaterials and Ag2O/RuO2 Nanocomposites
Source: Nanomaterials (Basel). 2016 Feb 26;6(3):42. doi: 10.3390/nano6030042 (PMC5302525; doi:10.3390/nano6030042)
Supplement: Supplementary file 1 [file nanomaterials-06-00042-s001.pdf]

Supplementary Materials: Dielectric Barrier Discharge (DBD) Plasma Assisted Synthesis of Ag_2O Nanomaterials and Ag_2O/RuO_2 Nanocomposites

Antony Ananth and Young Sun Mok*

1. Characterization Results of the RuO_2 Nanomaterials (NMs)

Figure S1a,b show the optical and field emission scanning electron microscope (FESEM) images of the RuO_2 NMs. The nanomaterials exhibited nanosheet morphology with length >1 µm and the width <100 nm. The X-ray diffraction (XRD) spectrum (Figure S1c) showed the prominent peaks at the diffraction angle (2θ) 28.00°, 34.93°, 39.64° and 54.00°, representing (110), (101), (200) and (211) set of lattice planes of rutile type RuO_2 (JCPDS card No.88-0322). The Thermogravimetric analysis (TGA) thermogram of the RuO_2 NMs (Figure S1d) showed the removal of oxygen in a gradual manner when increasing the temperature. Removal of the adsorbed water vapors until 120 °C, followed by gradual decomposition of RuO_x into metallic Ru at higher temperature was noted. The samples initially contained 24.04 wt % (10.338 mg) oxygen and the oxygen loss amounted to 22.84 wt % (9.822 mg). Still it contained about 1.192 wt % oxygen at 800 °C.

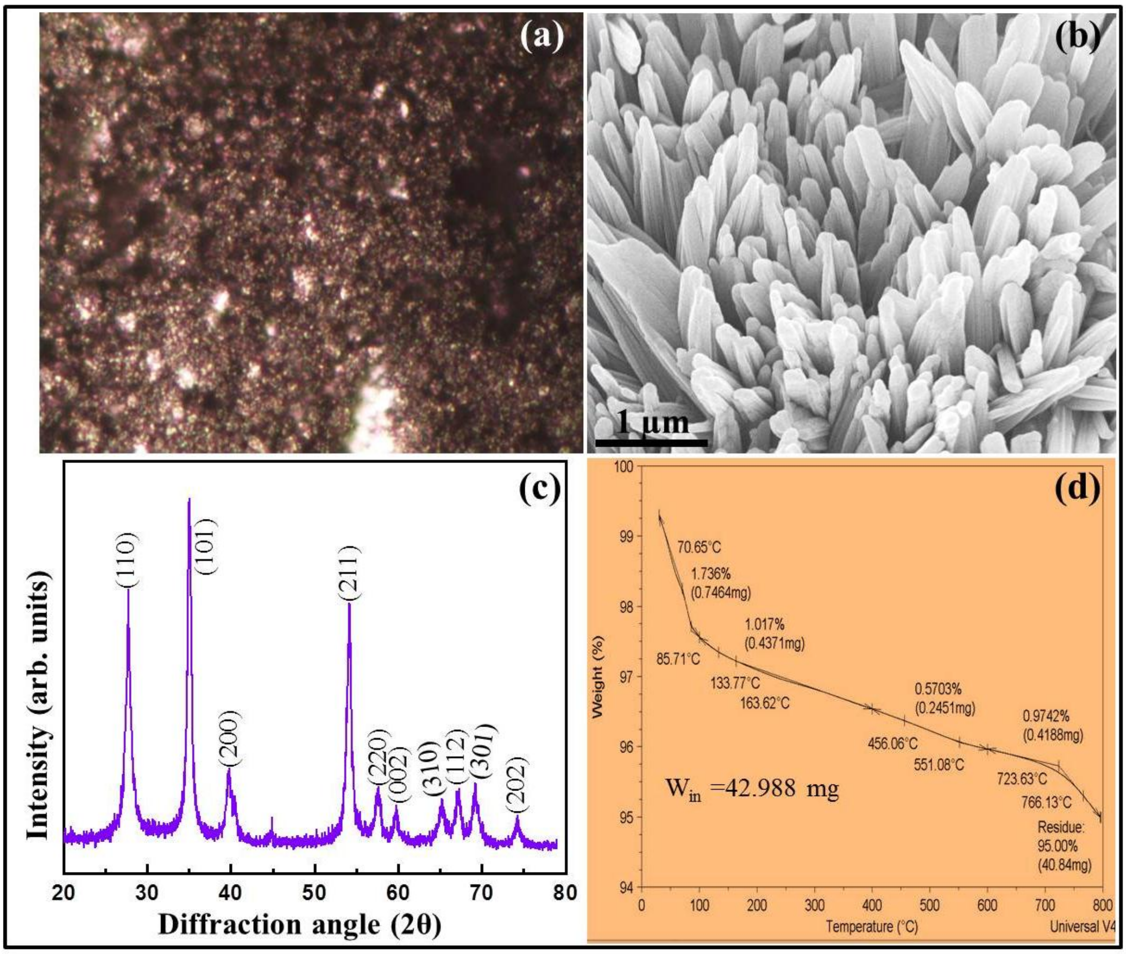

Figure S1. (**a**) Optical microscopic image at 100 times magnification; (**b**) The field emission scanning electron microscope (FESEM) image; (**c**) X-ray diffractogram; and (**d**) Thermogravimetric analysis (TGA) thermogram of RuO_2 Nanomaterials (NMs).

2. DBD Plasma Reactor Setup

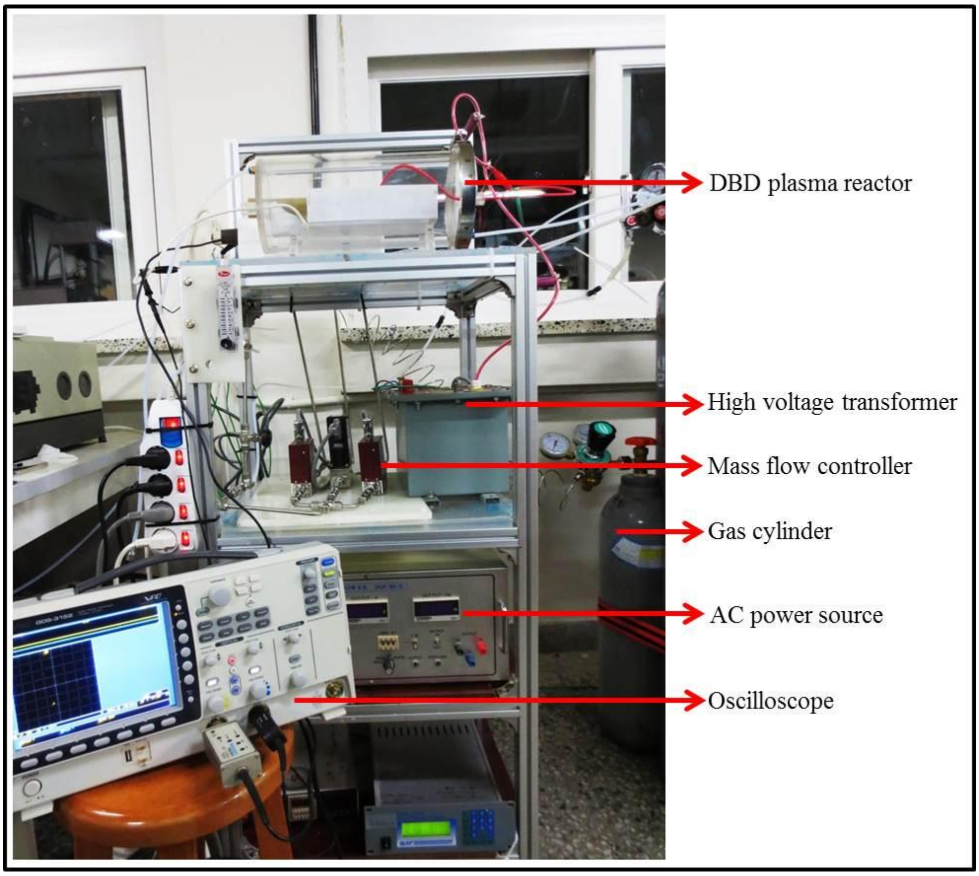

Figure S2. Photographic image of the Dielectric Barrier Discharge (DBD) plasma reactor.

© 2016 by the authors; licensee MDPI, Basel, Switzerland. This article is an open access article distributed under the terms and conditions of the Creative Commons by Attribution (CC-BY) license (http://creativecommons.org/licenses/by/4.0/).
